# Modeling the effects of grassland management intensity on biodiversity

**DOI:** 10.1002/ece3.6957

**Published:** 2020-11-03

**Authors:** Noëlle Klein, Coralie Theux, Raphaël Arlettaz, Alain Jacot, Jean‐Nicolas Pradervand

**Affiliations:** ^1^ Division of Conservation Biology, Institute of Ecology and Evolution University of Bern Bern Switzerland; ^2^ Agricultural Landscapes and Biodiversity, Department of Agroecology and Environment Agroscope Zürich Switzerland; ^3^ Department of Ecology and Evolution, Biophore University of Lausanne Lausanne Switzerland; ^4^ Swiss Ornithological Institute, Field Station Valais Sion Switzerland

**Keywords:** biodiversity, floral diversity, grassland management intensity, multitrophic, orthopteran abundance, plant indicator species, remote sensing

## Abstract

A growing food demand and advanced agricultural techniques increasingly affect farmland ecosystems, threatening invertebrate populations with cascading effects along the food chain upon insectivorous vertebrates. Supporting farmland biodiversity thus optimally requires the delineation of species hotspots at multiple trophic levels to prioritize conservation management. The goal of this study was to investigate the links between grassland management intensity and orthopteran density at the field scale and to upscale this information to the landscape in order to guide management action at landscape scale. More specifically, we investigated the relationships between grassland management intensity, floral indicator species, and orthopteran abundance in grasslands with different land use in the SW Swiss Alps. Field vegetation surveys of indicator plant species were used to generate a management intensity proxy, to which field assessments of orthopterans were related. Orthopteran abundance showed a hump‐shaped response to management intensity, with low values in intensified, nutrient‐rich grasslands and in nutrient‐poor, xeric grasslands, while it peaked in middle‐intensity grasslands. Combined with remote‐sensed data about grassland gross primary productivity, the above proxy was used to build landscape‐wide, spatially explicit projections of the potential distribution of orthopteran‐rich grasslands as possible foraging grounds for insectivorous vertebrates. This spatially explicit multitrophic approach enables the delineation of focal farmland areas in order to prioritize conservation action.

## Graphical abstract

Here, we investigate the links between grassland intensification processes and biodiversity on different trophic levels at the field and landscape scale in order to guide management actions at regional scale. The results of this spatially explicit multitrophic approach enable the delineation of focal farmland areas in order to prioritize conservation action.

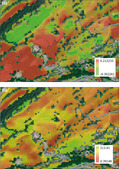

## 1 INTRODUCTION

A growing food demand and advances in farming technologies have led to intensified agricultural practices, that is, more efficient, industrial farming. This has caused massive losses of biodiversity in various ecosystems, with dramatic negative effects on ecosystem functions and services that are essential for human well‐being (Dirzo et al., 2014). Habitat loss and land conversion have thus been listed as the main drivers of biodiversity loss, along with fertilization and pesticide application (Sánchez‐Bayo & Wyckhuys, 2019). Land‐use changes lead to increased habitat homogeneity through the establishment of large intensively managed monocultures and removal of natural structures (Vickery & Arlettaz, 2012) which results in a decline of many animal taxa (Benton et al., 2003; Robinson & Sutherland, 2002). Extensively managed semi‐natural grasslands are particularly affected by land‐use changes and thus considered among the most threatened habitats in Europe (Canals & Sebastià, 2000). These grasslands are important habitats, hosting a high floral diversity and offering shelter for numerous endangered invertebrates and vertebrates that find their last refuges in these extensively managed habitats, such as insectivorous birds that have become rare (Knaus et al., 2018).

In the past decades, a severe global insect decline has been observed across many habitat types (Hallmann et al., 2017; Seibold et al., 2019). Intensified agricultural practices come along with increased irrigation and the application of herbicides, pesticides, and fertilizers. In intensively managed meadows, insect populations are affected either directly through insecticides (Goulson, 2013), mowing (Buri et al., 2016; Humbert et al., 2012), fertilization, and irrigation (Andrey et al., 2014), or indirectly through herbicides homogenizing plant diversity or limiting food resources (Robinson & Sutherland, 2002). Several studies have shown the negative impact of these intensification processes on vegetation structure (i.e., Cernusca et al., 1996) as well as on arthropod abundance, richness, and biomass (Lessard‐Therrien et al., 2018; Marini et al., 2008) indicative of a negative relationship between plant/insect species richness and grassland intensification. This does not only affect invertebrate communities but also cascades to animal groups depending on invertebrates as their food resources (Wilson et al., 1999). Therefore, the disappearance and degradation of extensively managed grasslands dramatically affects both faunal and floral biodiversity, underlining the importance of their preservation and necessary steps to take appropriate conservation action.

For a comprehensive protection and conservation planning of grassland biodiversity, we need strategies acting on multiple spatial scales. On the field scale, a fine understanding of the often‐complex relationships between intensification processes and floral and faunal diversity helps us adapting local management strategies such as mowing regimes and chemical inputs. In addition, effective complementary conservation strategies need to act on larger scales (landscape scale) in order to identify target key areas that ensure habitat and therefore population connectivity. However, to build such large‐scale conservation strategies, fine‐scaled field data are often not available or rarely cover large areas. This is why modeling tools such as species distribution models or essential biodiversity variables (Guisan et al., 2013; Kissling et al., 2018), which enable extrapolations of data on large scales are now increasingly used to complement these gaps. While some studies use stacked species distribution models or species richness models to prioritize conservation management areas (Vincent et al., 2019) major information on farming intensity and on species abundances—variables known to influence strongly species assemblages (Baudraz et al., 2018)—are often missing.

Remote sensing approaches are regularly used to distinguish semi‐natural from anthropogenic vegetation, to characterize ecosystem structure (grassland vs. shrubs) (Alleaume et al., 2018) as well as to produce a large‐scale assessment of grassland use intensity (Franke et al., 2012). These approaches are useful for the classification of grasslands into different land‐use categories, while not considering intracategorical variation. However, understanding this local intracategorical variation is of primary interest. Taking the example of extensively managed grasslands, the floral diversity they are hosting can shift dramatically depending on land use, insect diversity, and abundance despite being in the same meadow categories (Andrey et al., 2014; Buri et al., 2016). These fine scale phenomena are often not taken into account in modeling processes but are precious to identify areas of interest for conservation planning. With such high‐resolution quality maps, high quality areas could be prioritized for habitat preservation, while low quality areas can be incorporated into future habitat restoration actions. More fine‐scaled projections of local grassland management intensity, incorporating both floral and faunal diversity could serve as better tools for conservation planning and allow the delineation of high‐biodiversity grasslands at landscape scale.

The aim of this study was thus (a) to develop a proxy for management intensity based on vegetation surveys and remote sensing, to (b) investigate the complex links between grassland management intensity and floral and faunal biodiversity at field scale; and finally (c) to project this local information obtained from different trophic levels (vegetation and orthopterans) to the wider landscape providing a tool for local conservation management.

## 2 MATERIALS AND METHODS

### 2.1 Study area

The study was conducted from May to August 2018 in the SW Swiss Alps in the Canton of Valais (46°13'45.30"N 7°27'34.99"E) from an elevation of 500 to 1,400 m and thus ranging from the foothill belt to the beginning of the montane belt. A total of 57 meadows (Figure A.4, mean area 7,952 m^2^) located between Brig and Fully were selected for sampling. The meadow selection was randomized and balanced between the lowland and foothills, and the plots were equally distributed across the study area and along the elevation belts. The different plots are covering the whole gradient of intensification from natural steppe‐like grasslands to natural but intensively managed meadows and finally sown meadows.

On each of the 57 meadows, 10 random sampling points (for orthopteran sampling) were generated (random point tool (Quantum GIS Development Team 2018)). Among the 10 sampling points per site, three points (for vegetation surveys) were selected to fall inside distinct Sentinel‐2 pixels (10 x 10 m grid, provided by the Copernicus program led by the European Commission, processed at level 2A/3A by the CNES for the data center THEIA) on each meadow. To avoid clustering and edge effects (Knop et al., 2006), points which accumulated or met buildings, bushes, streets, trees, or edges were moved to the closest pixel of the grid. The normalized difference vegetation index (NDVI) was calculated from infrared and near‐infrared bands Sentinel‐2 as an estimation of primary production (Pettorelli et al., 2011) following the formula (NIR‐RED/NIR + RED). To avoid biased values, the pixels had to be free of confounding structures like bushes, trees, or buildings. NDVI was calculated for each month of the field sampling (April to August 2018).

### 2.2 Data collection

#### 2.2.1 Vegetation surveys

59 indicator plant species (Table A.1) were defined and considered representative for the predominant grasslands in the study area (Delarze et al., 2008). The presence and abundance of these different species vary along the intensification gradient allowing for a classification of the meadows from unmanaged grasslands to intensively managed grasslands. The species were chosen according to the definition of the different habitat types sensu Delarze et al. (2008), completed by the Swiss center for agricultural research species list used as an identification key to habitat types (Buholzer et al., 2015) and recommendations of botanists with local expertise (pers. com. P. Vittoz and A. Litsios‐Dubuis). Using a list of indicator species reduces drastically the sampling effort, maximizing thus the efficiency and the costs. It is thus commonly used by environmental offices. For example, such method is largely used to define grassland types in Switzerland (Delarze et al., 2008). In our case, we pooled and extracted from the existing lists the species from the local species pool.

Vegetation surveys were conducted on 3 vegetation plots (each a square of 4 m^2^, falling into distinct Senetinel‐2 pixels) per meadow between the 7th of May and 29th of July. The presence and abundance (% coverage) of all 59 indicator plant species was assessed to allow a gradual classification of each meadow according to different land‐use intensities. For a small number of meadows (*n* = 3), the vegetation was already mown at the first field visit; therefore, the vegetation surveys had to be conducted later in the season.

#### 2.2.2 Orthopteran sampling

On each of the 10 random points, orthopterans were sampled using a Biocenometer, which consists of a net fastened around a 1 m square hard circle, allowing to count the density of orthopterans on a standardized area (technique and sample size in line with Badenhausser et al., 2009; Humbert et al., 2012). All grasshoppers inside the device were counted and distinguished in categories (Table A.2). Orthopteran surveys took place in 4 sessions from 6th of June to 16th of August between 8:45 a.m. and 19:45 p.m., at a minimum temperature of 15 degrees and sunny weather (Pradervand et al., 2013). To avoid timing effects, sampling sites were randomized between each session. Additional variables were assessed in the field (Table 1).

**Table 1:** Explanatory variables which were used for the statistical analysis

Variable	Description	Multivariate models	
Month	June, July, August	Abundance	Richness
Mowing	If a meadow was mown (categorical: 0 = no, 1 = yes, 2 = second vegetation)	Abundance	Richness
Grazing	If a meadow was grazed (categorical, 0 = no, 2 = signs of former grazing, 3 = animals on a meadow)	Abundance	Richness
Irrigation	Irrigation or irrigation system installed (categorical, 0 = no, 1 = yes)	Abundance	Richness
Mean vegetation height	Height of vegetation on 1 m^2^ plot in [cm]	Abundance	Richness
Proportion of bare ground	Amount of bare ground 1 m^2^ plot in [%]	Abundance	
Sampling round	Sampling session (1, 2, 3, 4)		
Management intensity	Index for grassland management intensity, See Figure 1		
(Management intensity)^2^	Index for grassland intensification, See Figure 1	Abundance	Richness
GDD	Mean of growing degree days (GDD) above 3°C from 2000 to 2015, proxy of heat accumulation used to predict the development rates of plants (Zimmermann & Kienast, 1999)	Abundance	Richness

Note

The variables in italics were collected in the field, while the DCA management intensity proxy was derived from the analysis (linear and quadratic (^2^)), and the variable growing degree days was extracted from the WSL bioclim database (Broennimann, 2018). All variables except sampling session were included in the analysis. The last column lists all significant variables of the univariate models which were then included in the multivariate models.

### 2.3 Statistical analyses

#### 2.3.1 Estimating grassland management intensity

The ecology of all plant species of Switzerland is summarized by ecological and biological traits called Landolt values. These values are showing the multidimensional space favorable for a given species, describing its environmental niche (Landolt et al., 2010). We attributed to each indicator plant species its corresponding Landolt values, the ecological indicator values (EIVs). Among the different ecological values we selected; temperature (T), continentality (K), light preference (L), soil moisture variability (W), soil pH (R), nutrient content (N), and soil aeration (D) for the analyses.

T indicates temperature preference and is highly correlated with temperature from climate models (Scherrer & Guisan, 2019), expressed in a gradient 1–5 from cold‐indicator to warmth‐indicator. L is associated with plant light preference and expressed in a gradient 1–5 from shaded to sunny areas. K indicates continentality, associated with plant distributions (Descombes et al., 2020), and expressed in a gradient 1–5 from atlantic to continental climates. W is indicative of soil moisture variability and expressed in a gradient 1–3 from low to high intra‐annual variability in soil moisture. R indicates soil pH, expressed as a gradient 1–5 from acidic to alkaline soils. N is indicative for soil nutrients (i.e., nitrogen, phosphorus) and expressed in a gradient 1–5 from nutrient‐poor to nutrient‐rich soils. D indicates soil aeration (oxygen supply) and is associated with hydromorphology (Descombes et al., 2020), expressed in a gradient 1–5 from waterlogged/low‐aerated soil to rocky/sandy soil. These indicator values have been shown valuable for assessing soil and climatic conditions, even outperforming traditional broad‐scale topoclimatic predictors such as temperature, precipitation, moisture, or pH proxies (Descombes et al., 2020), as EIVs taken from local communities give information on much more local conditions than other measures.

For each vegetation plot, a mean Landolt value per variable was computed by averaging the respective Landolt values of all species present on a plot and accounting for their relative abundance, following Dubuis et al. (2013). A Detrended Correspondence Analysis (DCA, (Hill & Gauch, 1980)) was conducted in R (R Core Team, 2018), using the *decorana* function of the *vegan* R package (Oksanen et al., 2018) to test whether the computed average Landolt values can be used as a proxy for management intensity on each meadow. We then used the value from the first axis of the DCA for each plot, as this axis is strongly shaped by nutrient content (N) and soil aeration (D) thus representative of the intensification gradient. The final meadow value resulted in the average DCA value of the three plots per meadow.

#### 2.3.2 Orthopteran analysis

To understand which variables were influencing orthopteran abundance and orthopteran species richness, we used two model selection approaches with these variables as responses. Orthopteran abundance was defined as the sum of orthopteran individuals per plot (including nymphs). The number of species groups was considered as a proxy for species richness, as it has been shown that higher taxa diversity can be used as a surrogate for species diversity (Báldi, 2003; Balmford et al., 1996). For the categories which had not been identified to species level, the genus was considered a species group (e.g., Chorthippus, see Table A.2), providing a conservative measure of species richness. Nymphs were excluded from this part of the analysis, as they could not always be attributed to species groups.

The explanatory variables were management intensity and collected in the field; month, and survey session, bare ground, mean vegetation height, irrigation, grazing, mowing, and an interaction between month and (management intensity)^2^ (Table 1). In addition, the bioclimatic variables precipitation (averaged period: 1981–2010) and the mean of growing degree days (GDD, a proxy of heat accumulation above 3°C from 2000 to 2015, used to predict the development rates of plants (computed following Zimmermann & Kienast, 1999)) were extracted for each sampling point from (Broennimann, 2018). As the data were on a 25 x 25 m grid resolution, we resampled it to a 10 x 10 m resolution using spline interpolations, fitting the NDVI grid cells.

All variables were analyzed on plot scale (1 m^2^). The response variables had poisson distributions and showed no signs of zero‐inflation (*DHARMa* package in R (Hartig, 2019)). The model analyses were performed with generalized linear mixed models (GLMM) using the function *glmer* of the *lme4* R package (Bates et al., 2015). The meadow ID and sampling plot ID were included as random factors. In a first step, univariate models were performed on all explanatory variables (linear and quadratic (^2^), with collinearity lrl < 0.7 (Figure A.5), Spearman rank correlation tests, (Dormann et al., 2013)) and the 11 (abundance), respectively, 10 (richness) best variables were included in the further analyses (Table A.3). The response variables were standardized (*mutate_at* function of *dplyr* R package (Wickham et al., 2018)) to improve model convergence. Multivariate models were then performed and ranked by Akaike information criterion (AIC) with the *dredge* function of the multimodel inference (*MuMIn*) R package (Barton, 2018). Overdispersion of count data was tested with the *dispers_glmer* function of the *blmeco* R package (Korner‐Nievergelt et al., 2015) and accounted for by including observation‐level random effects (Harrison, 2014). Finally, all models with Δ AIC <2 were used to compute an averaged best model and spatial autocorrelation was tested for with a mantel test.

All response relationships were produced by using the *sim* function of the *arm* R package (Gelman et al., 2018), which is simulating the response of the averaged best model by drawing samples from joint posterior distributions.

#### 2.3.3 Projection on the landscape scale

In order to obtain high‐resolution maps of management intensity and orthopteran abundance at landscape scale, new models were built, incorporating additional variables on a broader spatial scale. As local variables (such as irrigation, grazing, mowing, bare ground, vegetation height, etc.) are not available or possible to sample on large contiguous areas, we projected management intensity and orthopteran abundance using a set of variables available for the whole area. The model projections were carried out in two steps. The first step was to use NDVI calculated from the Sentinel data to project management intensity on the landscape scale, while the second was to use this prediction to further project orthopteran abundance on the landscape scale of the whole Canton of Valais (548,578.4, 679,788.4, 87,708.77, 167,428.8, open vegetation areas below 1,400 m elevation).

Univariate models were performed on NDVI variables from April to August 2018 (year of sampling), distance to bushes, distance to forest, mean temperature, mean precipitation, growing degree days, aspect, slope, curvature, and solar radiation (computed following method by (Zimmermann & Kienast, 1999), with collinearity lrl <0.7, Spearman rank correlation tests, (Dormann et al., 2013)). The best variables (*p* values <.05) were included in the final models with management intensity and orthopteran abundance as response variables. Seven outliers were excluded from further analyses as they were traced back to confounding structures in the NDVI grid cells. The only significant way to model management intensity was a linear model (lm) using the DCA1 output (DCA1, unit: vegetation plot) based on NDVI of April 2018 as explanatory variable. A generalized linear model (glm, Ortho ~ management intensity projection) was used to predict orthopteran abundance at landscape scale, based on the previously projected management intensity proxy. Orthopteran abundance was predicted as the mean orthopteran sum per meadow to account for variation caused by microscale effects (as shown in 3.2.) which cannot be included as model predictors.

The accuracy of both models was assessed using repeated cross‐validation, the most commonly used method when independent datasets are not available (Guisan & Zimmermann, 2000), and for the management projection, we randomly selected 1,000 times 80% of the data (weighted by habitat type sample size) as a training dataset and predicted the model on the remaining 20%. For cross‐validation of the orthopteran projection, 70% were used for training and 30% for validation, accounting for a small sample size (Fielding & Bell, 1997). In addition, we randomly drew 100 points (avoiding confounding structures like trees/bushes) falling inside the Swiss inventory of dry meadows and pastures—thus extensive to very extensive meadows—(Bundesamt für Umwelt, 2017) and compared the predicted DCA1 values with this independent dataset.

## 3 RESULTS

### 3.1 Vegetation

**Figure 1:**
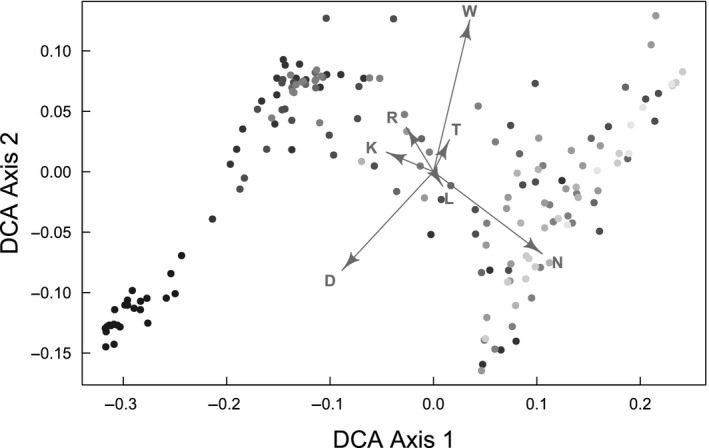
Detrended correspondence analysis (DCA) for the mean Landolt values per plot, computed from field vegetation surveys. Grayness illustrates the coverage weighted values of the indicator plant species (dark = more extensive), which are linked to differential land use according to Buholzer et al. (2015) and Delarze et al. (2008). Therefore, DCA1 shows the management intensity gradient from zero‐low and low‐medium management intensity on the left to high‐medium and very high management intensity on the right. The arrows correspond to the Landolt values T (Temperature), K (Continentality), L (Light preference), W (Soil moisture variability), R (Reaction), *N* (Nutrient content), D (Soil aeration) and illustrate how they are driving the DCA axes.

The DCA was shown to distinguish the vegetation plots according to the predefined grassland land use following indicator plant species (Figure 1). DCA axis 1 explained 76.43% of all variance and fitted the gradient of management intensity. The first two axes together explain 95.43% of the variance in the data, and both are driven by a combination of different Landolt values (see Figure 1). Therefore, the DCA axis 1 value was attributed to each vegetation plot. This value was then used as a management intensity proxy ranging from low values for low‐zero management intensity to high values for very high management intensity (artificial, i.e. sown meadows).

### 3.2 Orthopterans

A total of 8,725 orthopteran individuals were counted with the biocenometer sampling.

**Figure 2:**
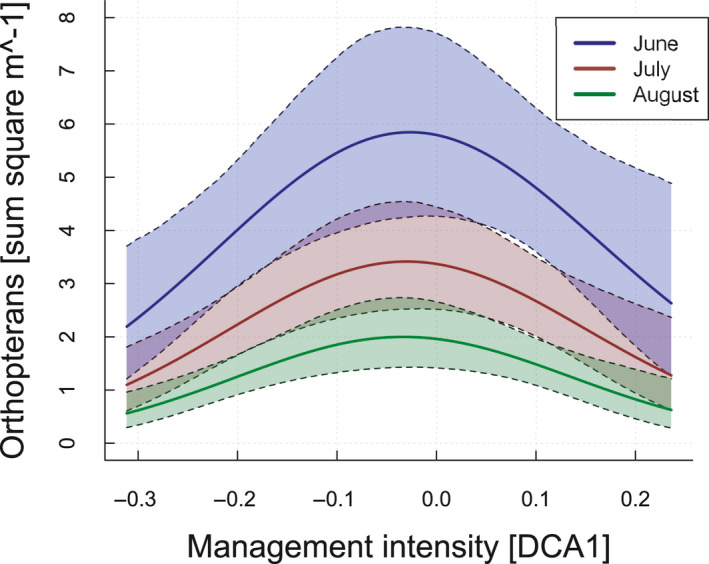
Orthopteran abundance in relation to the management intensity index (see Figure 1).

**Figure 3:**
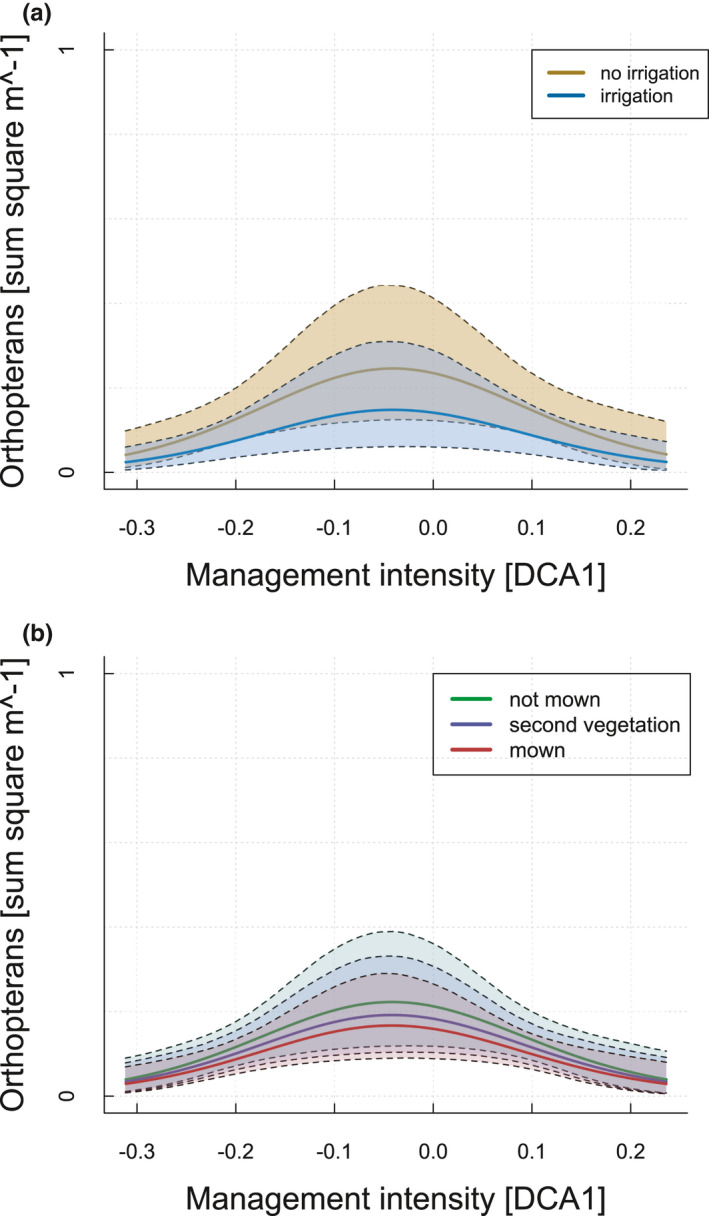
(a,b) Orthopteran abundance in relation to the management intensity index (see Figure 1). Colored lines show different (a) irrigation and (b) mowing states (second vegetation = regrown after mowing)

According to univariate models, eight environmental variables affected orthopteran abundance (Table A.3). Including these variables in the model selection approach, three models were competitive with a Δ AIC < 2 (Table A.4). The conditional averaged model (Table 2) showed that orthopteran abundance had a hump‐shaped relationship with management intensity (−0.34 ± 0.10, z = 3.31, *p* < .001; Figure 2). Furthermore, orthopteran abundance decreased with higher amount of bare ground (−0.17 ± 0.03, z = 6.22, *p* < .001), increasing growing degree days (−0.29 ± 0.10, z = 2.87, *p* > .01), presence of irrigation (−0.27 ± 0.11, z = 2.38, *p* < .05; Figure 3a), month (−0.41 ± 0.02, z = 14.31, *p* < .001), and mowing (second vegetation = regrown after mowing: −0.32 ± 0.09, z = 3.34, *p* < .001/ freshly mown: −0.23 ± 0.10, z = 2.36, *p* < .05; Figure 3b). In addition, there was a significant interaction between the degree of intensification and month on orthopteran abundance (−0.05 ± 0.02, z = 2.01, *p* = .05). Visual inspection indicates that the hump‐shaped relationship was most pronounced in June and became weaker later in the season. There was no sign for spatial autocorrelation in the orthopteran data (Mantel test, *p* > .05).

**Table 2:** Summary of the model‐averaged coefficients (conditional average) of all models with Δ AIC < 2 (see Table A.4) investigating orthopteran abundance and richness

Model	Variables	Var. importance	Estimate ± SE	z value	p value
Orthopteran abundance	Management intensity	1.00	0.11 ± 0.10	1.09	ns (<0.5)
	**Management intensity^2^**	1.00	−0.34 ± 0.10	3.31	<0.001
	**Month**	1.00	−0.41 ± 0.02	14.31	<0.001
	Bare ground	1.00	−0.06 ± 0.03	1.79	ns (<0.1)
	**Bare ground^2^**	0.90	−0.17 ± 0.03	6.22	<0.001
	Vegetation height	1.00	0.04 ± 0.03	1.24	ns (<0.5)
	**Vegetation height^2^**	1.00	−0.20 ± 0.03	6.88	<0.001
	**GDD**	0.96	−0.29 ± 0.10	2.87	<0.01
	Grazing2 (formerly)	0.47	−0.10 ± 0.11	0.87	ns (<0.5)
	Grazing3 (yes)		0.14 ± 0.13	1.02	ns (<0.5)
	**Irrigation**	0.87	−0.27 ± 0.11	2.38	<0.05
	**Mowing (freshly mown)**	0.99	−0.23 ± 0.10	2.36	<0.05
	**Mowing (2nd vegetation)**		−0.32 ± 0.09	3.34	<0.001
	**(Management intensity)^2^ x Month**	0.79	−0.05 ± 0.02	2.01	<0.05
Orthopteran species richness	Management intensity	1.00	0.15 ± 0.08	1.89	ns (<0.1)
	**Management intensity^2^**	1.00	−0.29 ± 0.07	3.90	<0.001
	**Month**	1.00	0.20 ± 0.03	6.95	<0.001
	Bare ground	0.95	−0.03 ± 0.04	0.73	ns (<0.5)
	**Bare ground^2^**	0.95	−0.09 ± 0.03	2.97	<0.01
	**Vegetation height**	1.00	−0.08 ± 0.04	2.14	<0.05
	**Vegetation height^2^**	1.00	−0.16 ± 0.04	4.13	<0.001
	GDD	0.60	−0.12 ± 0.07	1.72	ns (<0.1)
	**Grazing (formerly)**	1.00	0.39 ± 0.11	3.55	<0.001
	Grazing (yes)		0.11 ± 0.14	0.77	ns (<0.5)
	Mowing (freshly mown)	1.00	0.16 ± 0.10	1.66	ns (<0.1)
	**Mowing (2nd vegetation)**		−0.26 ± 0.10	2.53	<0.05
	(Management intensity)^2^ x Month	0.29	0.02 ± 0.02	0.64	ns (<1)

Note

All variables (significant ones shown in bold, (linear and quadratic (^2^))), their relative importance in the averaged model and estimates with standard error, z value, and *p* value are shown (ns, not significant, *p *> .05).

Species richness was affected by eight environmental variables according to univariate models (Table A.3). Including these variables in the model selection approach, three models were competitive with a Δ AIC < 2 (Table A.4). The conditional averaged model (Table 2) showed that orthopteran species richness had a hump‐shaped relation with the degree of intensification (−0.29 ± 0.07, z = 3.90, *p* < .001; Figure A.2) and was negatively affected with vegetation regrowth (second vegetation: −0.26 ± 0.10, z = 2.53, *p* < .05), bare ground (−0.09 ± 0.03, z = 3.97, *p* < .01), and mean vegetation height (−0.16 ± 0.04, z = 2.14, *p* < .05). In contrast, species richness increased with month (0.20 ± 0.03, z = 3.90, *p* < .001) and previous grazing (formerly: 0.39 ± 0.11, z = 3.55, *p* > .001).

### 3.3 Projection on the landscape scale

Among the different variables available at the landscape scale, namely NDVI variables from April to August 2018 (year of sampling), distance to bushes, distance to forest, mean temperature, mean precipitation, growing degree days, solar radiation, aspect, slope, and curvature, only NDVI was significantly linked to the management intensity. We thus used it for the projection as linear model DCA1 ~ NDVI April 2018 (0.79 ± 0.04, *p* < .001, Figure A.4). When performing a habitat‐weighted repeated cross‐validation for the vegetation projection, a correlation of 86.27% (*p* < .001) was obtained, indicating a high accuracy of the model. The model performed thus very well in predicting management intensity (Figure A.3a), while there is a weak overprediction on the intensive part of the gradient between 0.05 and 0.2. In addition, the model proofed accurate when drawing 100 random points from the dry meadow inventory, which yielded mean predicted DCA1 values of −1.89 ± 0.08 (from −0.30 to 0.08), therefore fitting the expected values for extensive meadows from our management projection. In total, the projection predicts 29.97% of the study area (total 247,900 ha) to be covered by grassland, of which 34.19% (10.25% of the study area) is attributed to high‐medium management intensity, 32.25% (9.64% of the study area) to medium‐low management intensity, 24.58% (7.37% of the study area) to very high management intensity and 8.99% (2.69% of the study area) to low‐zero management intensity.

**Figure 4:**
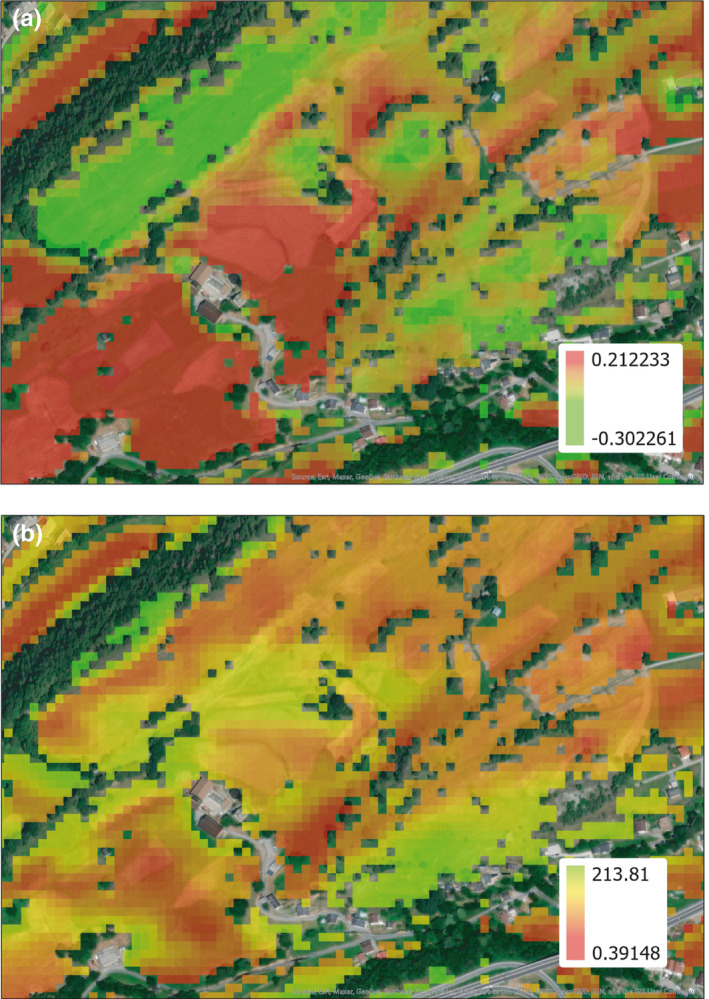
(a) Model projection map showing the extrapolated management intensity index to the whole landscape. Green areas are predicted to be more extensively managed than red areas which are predicted to be intensively managed. (b) Model projection map showing the extrapolated orthopteran density (as drawn from the intensification index map, Figure 1). Green areas are predicted to harbor a higher density of orthopterans than red areas.

Similarly, orthopteran abundance at field scale was affected by management intensity (quadratic) and solar radiation (quadratic). Orthopteran abundance was projected as generalized linear model Ortho ~ management intensity (−3.86 ± 2.53, *p* < .2) + management intensity^2^ (−4.28*10^1^ ± 1.35, *p* < .01) + Solar radiation (−1.22*10^−2^ ± 3.83*10^−3^, *p* < .01) + Solar radiation^2^ (2.44*10^−7^ ± 7.41*10^−8^, *p* < .01). When performing repeated habitat‐type weighted cross‐validation for the orthopteran projection, a correlation of *r* = 40.15% (*p* < .001) was obtained. The model performed well in predicting low to average values but still with an important error rate (Figure A.3b). A few points showing a high orthopteran abundance like the three outliers from Figure A.3b hosting more than 10 orthopterans show an important underprediction. See Figure 4 for maps with the projected management intensification (a) and orthopteran abundance (b), Figure A.6 for residuals and the spatial distribution of the prediction error.

## 4 DISCUSSION

First, using vegetation surveys to infer management intensity, set in relation to orthopteran abundance, we demonstrate an optimum in traditionally managed meadows. Second, by upscaling to the landscape, grasslands of high importance could be spatially delineated, delivering an important tool for large‐scale conservation planning. In the following sections, we will discuss the management intensity proxy, the relationship of different environmental and management‐related factors with orthopteran abundance and richness, and finally our projections to the landscape scale.

Vegetation surveys are necessary to assess habitat quality at local scale. While grassland is often only classified into broad land‐use categories, our method incorporates intracategorical variation by allocating a field‐data based degree of management intensity to each meadow. This proxy acts as basis for a more fine‐scaled analysis of the relationships between land use and insect abundance and diversity.

Orthopteran abundance and richness showed a quadratic relationship to grassland management intensity, with low values in either high‐medium and low‐zero intensity meadows and an optimum in medium‐low intensity meadows. The highest orthopteran abundance and richness were found in *Mesobromion* meadows, traditionally managed grasslands known to harbor a high diversity of plant species (Delarze et al., 2008). These *Mesobromion* meadows nowadays only cover about 1% of the agricultural area of Switzerland and have been dramatically declining following agricultural intensification, with losses of up to 90% of their historical area since 1945 (Eggenberg et al. 2001; Masé, 2005). In Valais, our results indicate that there is still a very high proportion of these meadows with 32.25% of the projected grassland area and 9.64% of the projected study area, illustrating the high responsibility of this region for the conservation of this kind of grasslands and its associated biodiversity. Their importance for orthopterans and related predators also makes them most effective for conservation efforts (Kleijn et al., 2012), while their preservation is essential, as they have been predicted to disappear without active counter‐measures (Masé, 2005). The low values of orthopterans in intensified grasslands are in line with previous studies (e.g. Delley, 2014) as several known factors were shown to negatively affect orthopterans. Human disturbance has been shown to affect insects (Kati et al., 2012), while an important factor for orthopteran mortality is mowing, as orthopterans are either killed through the mechanical process or exposed to predators due to low grass height (Arlettaz, 1996) or flee to unmown refuges, thereby decreasing orthopteran abundance on the meadow (Buri et al., 2016; Humbert et al., 2012). After regrowth of the vegetation, orthopteran abundance increased again without reaching the initial values. Frequent mowing can be associated with intensively managed meadows that are heavily fertilized and irrigated. In line with this and former studies (Andrey et al., 2014), irrigation had a negative effect on orthopteran abundance and richness. This finding is illustrated by the quadratic relationship with management intensity and shows that traditional low‐medium intensity management is associated with higher orthopteran abundance and richness compared with very extensive grassland habitats (*Stipo‐Poion*) that are rarely managed, but harbor a low orthopteran abundance and richness. *Stipo‐Poion* is listed as grassland of international importance, not because of its high insect abundance but because it is occupied by floral and faunal specialists of xeric and warm habitats (Delarze et al., 2008; Masé, 2005). Linked to this, there was a negative effect of the amount of bare ground on orthopteran abundance, as steppes are a habitat with extreme conditions and much bare ground, typically occupied by specialists (Delarze et al., 2008; Masé, 2005) such as *Calliptamus italicus* and *Oedipoda spp*. (Baur et al., 2006). Overall, orthopteran abundance was shown to decrease throughout the reproductive season, while species richness increased. In the early season, a high abundance of nymphs explains the high orthopteran abundance, while most of the nymphs are doomed to die before reaching maturity. Species richness was shown to increase after mowing, supporting the role played by seasonality, as phenology leads to higher species richness in July and August compared with June. In addition, grazing was shown to have positive effects on species richness, as semi‐extensive meadows which harbor a higher diversity of species groups are often grazed (Delarze et al., 2008).

We show how diverse environmental and management factors influence orthopterans at the field scale. The findings illustrate the negative effects of land use and management intensification on orthopterans, in line with the current drastic declines of insect populations (e.g. Seibold et al., 2019) and previous studies on land‐use change (Hofstetter et al., 2015). Previous studies have also shown dramatic population declines of higher trophic levels, which depend on insects as a food resource (Bowler et al., 2019). These declines are especially drastic for insectivorous farmland birds, relying on grassland (Bowler et al., 2019), suggesting a link to land‐use intensification processes and the disappearance of traditionally managed grasslands.

While modeling approaches are often dealing with species distributions or richness, we often miss proxy for taxa abundances. Such data are however crucial when conservation targets insectivorous predators which depend on food resources. Our approach on the projection of floral and faunal diversity proxies provides a new and efficient technique for the assessment of different trophic levels of diversity and abundance on a landscape scale. The management intensity proxy was highly correlated to NDVI and therefore allowed a very accurate projection at landscape scale. The further projection of orthopteran abundance performed less accurately but still explained nearly half of the variance in the overall data, providing a solid tool for the delineation of priority grasslands, even highlighting small scale differences in management, like uncut refuges along mown meadows. The shortcomings of our projections are, first, the dependency of the second projection on the first, which comes along with an error accumulation and a drop of explained variance. Second, several microscale factors (see 3.2) were shown to influence orthopterans, but could not be included in the projections at landscape scale, due to the impossibility to record these data at that scale; therefore, orthopteran values had to be averaged per meadow. The high correlation of orthopterans and the intensification index with NDVI and the absence of significant response of the topoclimatic variables are probably indicating that at the scale of our study, land‐use changes are more important than topoclimatic drivers like (Baudraz et al., 2018) showed in the Prealps. Thus, the main limitation of the projections is linked to the concept of remote sensing, which always leads to simplification through averaging values on pixel grids, thereby causing information loss with regard to meadow heterogeneity, confounding structures, and especially microscale effects, which are limited to the field scale. This partly explains the underprediction of the orthopteran count as orthopteran distribution can vary strongly within a single meadow due to small undetectable structures. These microscale effects that cannot really be assessed remotely with our approach include landscape characteristics (Le Provost et al., 2017) such as grassland cover, vegetation height, bare ground, irrigation, and dryness, but also ecological links where specific plant species are preferred as food resources (Schaffers et al., 2008). However, our new multiscale approach to project our land‐use management intensity proxy on a landscape scale by combining vegetation surveys with remote sensing data, and then in turn with orthopteran data, has to be considered as a first step to highlight valuable objects for biodiversity on which the results of the fine scale analysis can then be applied. The technique is time and labor effective and provides a valuable method to build prediction maps of grassland management and orthopteran abundance that can be used as a tool for applied conservation management. On the one hand, grasslands of excellent quality (which we show are still prevalent in Valais) can be prioritized for conservation, notably by promoting extensive management practices on these areas. On the other hand, grasslands of lower quality can be prioritized for habitat restoration: for instance, habitat quality can be improved if these areas are declared and subsidized as biodiversity promotion areas (wildflower strips or unmown refuges, etc.). To that aim, our fine scale results provide a promising approach not only for the delineation of focal areas but also for specifying which measures should optimally be applied.

## 5 CONCLUSIONS

In the framework of the current biodiversity decline, our results highlight the importance of traditionally managed low‐middle intensity meadows and show that orthopteran abundance and richness are highly affected by agricultural management and land‐use processes. This illustrates the importance of conservation measures linked not only to the preservation but also to the restoration of semi‐extensive grassland habitats in order to retain a high abundance and diversity of orthopterans. If not considered an end in itself, orthopterans are a very important resource for higher trophic levels such as insectivorous birds and have to be maintained in order to protect insectivores that currently count among the most impacted biodiversity taxa. Operating with field‐collected information about vegetation intensification and prey abundance while upscaling with an informative proxy we were able to build an efficient tool to highlight and prioritize focal areas for grassland biodiversity conservation and restoration in an inner Alpine valley.

## CONFLICT OF INTERESTS

The authors declare no competing interests.

## AUTHOR CONTRIBUTIONS

**Noëlle Klein:** Data curation (lead); Formal analysis (lead); Investigation (equal); Project administration (equal); Software (equal); Visualization (lead); Writing‐original draft (lead); Writing‐review & editing (equal). **Coralie Theux:** Data curation (equal); Formal analysis (supporting); Investigation (equal); Software (supporting); Writing‐review & editing (equal). **Raphaël Arlettaz:** Supervision (supporting); Writing‐review & editing (equal). **Alain Jacot:** Conceptualization (equal); Formal analysis (supporting); Methodology (equal); Project administration (equal); Software (supporting); Supervision (equal); Writing‐original draft (supporting); Writing‐review & editing (equal). **Jean‐Nicolas Pradervand:** Conceptualization (equal); Formal analysis (supporting); Methodology (equal); Project administration (equal); Resources (lead); Software (supporting); Supervision (lead); Writing‐original draft (supporting); Writing‐review & editing (equal).

## Supporting information

Appendix S1

## Data Availability

The complete data (field data of Vegetation surveys and Orthopteran counts and explanatory variables, as well as R codes of the analyses) is being archived at the data depository of the Swiss Ornithological Institute and available upon request.
