# Supplementary material for: Modeling the effects of grassland management intensity on biodiversity
Source: Ecol Evol. 2020 Nov 3;10(23):13518–29. doi: 10.1002/ece3.6957 (PMC7713963; doi:10.1002/ece3.6957)
Supplement: Supplementary file 1 — Appendix S1 [file ECE3-10-13518-s001.docx]

**Supporting information**

**Table A.1.:** List of all indicator species (or groups) considered for the vegetation surveys and analysis.

| Habitat | Abbreviation | Indicator species |
| --- | --- | --- |
| Arrhenatherion | *Arr* | *Arrhenatherum elatius*  *Dactylis glomerata*  *Crepis biennis*  *Knautia arvensis*  *Ranunculus acris*  *Anthriscus sylvestris*  *Galium album*  *Trifolium repens*  *Trifolium thalii (high alt.)*  *Heracleum sphondylium*  *Veronica chamaedrys*  *Trisetum flavescens*  *Holcus lanatus*  *Rumex acetosa*  *Bromus hordeaceus*  *Cynosurus cristatus*  *Festuca pratensis*  *Taraxacum officinale*  *Anthoxanthum odoratum*  *Rhinanthus alectorolophus* |
| Mesobromion | *Meso* | *Bromus erectus*  *Salvia pratensis*  *Brachypodium pinnatum*  *Hippocrepis comosa*  *Carex caryophyllea*  *Anthyllis vulneraria*  *Onobrychis viciifolia*  *Potentilla neumanniana*  *Scabiosa columbaria*  *Thymus serpyllum*  *Ononis repens*  *Gallium verum*  *Helianthemum nummularium*  *Koeleria pyramidata*  *Pimpinella saxifraga*  *Ranunculus bulbosus*  *Trifolium montanum*  *Daucus carota*  *Sanguisorba minor*  *Euphorbia cyparissias* |
| Stipo-Poion | *Steppe* | *Astragalus onobrychis*  *Scabiosa trianda*  *Silene otites*  *Artemisia campestris*  *Onobrychis arenaria*  *Erysimum rhaeticum*  *Euphorbia seguieriana*  *Potentilla pusilla*  *Stipa capillata*  *Stipa pennata*  *Carex liparocarpos*  *Poa perconcinna*  *Pulsatilla montana*  *Scorzonera austriaca* |
| Artificial | *Intensive* | *Lolium sp. (multiflorum/ perenne)*  *Alopecurus pratensis*  *Poa sp. (pratensis/ trivialis)*  *Trifolium pratense*  *Medicago sativa* |

**Table A.2.:** Table with all orthopteran categories and the sum of observed orthopterans per category. * indicate that the category was included as a species group in the response variable “species richness”.

| Category | Count |
| --- | --- |
| *Caelifera nymphs (<1.5cm)* | 4199 |
| *Caelifera nymphs (>1.5cm)* | 1579 |
| *Chorthippus all sp.** | 1317 |
| *Pseudochorthippus parallelus (subset of C. all)* | 333 |
| *Stenobothrus sp.** | 166 |
| *Ensifera big other nymphs* | 158 |
| *Calliptamus italicus** | 118 |
| *Platycleis albopunctata** | 116 |
| *Stauroderus scalaris** | 114 |
| *Gryllus campestris nymph** | 107 |
| *Ensifera big green nymphs** | 69 |
| *Platycleis albopunctata nymph** | 56 |
| *Tetrix sp.** | 50 |
| *Ensifera small nymphs (<1.5cm)* | 46 |
| *Mecostethus parapleurus** | 40 |
| *Roesiliana roeselii nymph** | 38 |
| *Mantis religiosa** | 37 |
| *Roesiliana roeselii** | 32 |
| *Nemobius sylvestris** | 31 |
| *Tettigonia viridissima** | 24 |
| *Euthystira brachyptera** | 24 |
| *Arcyptera fusca** | 14 |
| *Oedipoda sp.** | 17 |
| *Oecanthus pellucens** | 17 |
| *Decticus verrucivorus** | 16 |
| *Leptophyes punctatissima** | 10 |
| *Pholidoptera sp.** | 10 |
| *Phaneroptera sp.** | 8 |
| *Gryllus campestris** | 8 |
| *Conocephalus fuscus** | 2 |

**Table A.3:** Summary of the univariate mixed models on the effect of management intensity, Month, Bare ground, Irrigation, Mowing, Grazing and GDD on orthopteran abundance (glmer) and species richness (glmer). Model number, variables, estimates with standard error, z/t value and p value are listed (ns = not significant, p>0.05).

| Response variable | Model | Explanatory variable (univariate) | Estimate ± SE | z/t value | p value |
| --- | --- | --- | --- | --- | --- |
| *Orthopteran abundance* | 1 | Management intensity  Management intensity^2^ | -0.03±0.11  -0.39±0.11 | -0.29  -3.58 | ns (<1)  <0.001 |
|  | 2 | Month | -0.65±0.02 | -40.20 | <0.001 |
|  | 3 | Bare ground | -17.82±0.80 | -22.33 | <0.001 |
|  |  | Bare ground^2^ | -3.60±0.74 | -4.85 | <0.001 |
|  | 4 | Vegetation height  Vegetation height^2^ | 12.70±0.61  -11.72±0.72 | 20.96  -16.22 | <0.001  <0.001 |
|  | 5 | Irrigation | -0.19±0.06 | -3.30 | <0.001 |
|  | 6 | Mowing (freshly mown)  Mowing (second vegetation) | -0.95±0.04  -0.73±0.03 | -23.94  -20.18 | <0.001  <0.001 |
|  | 7 | Grazing (formerly)  Grazing (yes) | -0.21±0.05  0.30±0.06 | -3.95  4.77 | <0.001  <0.001 |
|  | 8 | GDD | -0.34±0.11 | -3.01 | <0.01 |
| *Orthopteran species* | 9 | Management intensity  Management intensity^2^ | 0.13±0.08  -0.31±0.08 | 1.75  -4.07 | <0.01  <0.001 |
| *richness* | 10 | Month | 0.35±0.03 | 11.43 | <0.001 |
|  | 11 | Bare ground | -1.44±1.35 | -1.07 | ns (<0.5) |
|  |  | Bare ground^2^ | -4.53±1.24 | -3.67 | <0.001 |
|  | 12 | Vegetation height  Vegetation height^2^ | -6.50±1.45  -10.56±1.75 | -4.50  -6.03 | <0.001  <0.001 |
|  | 13 | Irrigation | -0.20±0.11 | -1.85 | ns (<0.1) |
|  | 14 | Mowing (freshly mown)  Mowing (second vegetation) | 0.50±0.07  -0.13±0.07 | 7.21  -1.84 | <0.001  ns (<0.1) |
|  | 15 | Grazing (formerly)  Grazing (yes) | 0.52±0.10  0.09±0.13 | 5.32  0.66 | <0.001  ns (<0.1) |
|  | 16 | GDD | -0.18±0.08 | -2.18 | <0.05 |

**Table A.4:** Model selection table showing all models with $\Delta$AIC<2, all variables retained, degrees of freedom, the difference of AIC compared to the first and best model and the Akaike weight. X depicts an interaction term between DCA1.q and Month.

| Response |  | Model | Variables | df | $\boldsymbol{\Delta}$AIC | Akaike weight |
| --- | --- | --- | --- | --- | --- | --- |
| *Orthopteran abundance* |  | 4064 | Bare ground + Bare ground^2^+ Management intensity + Management intensity^2^+ GDD + Irrigation + Month + Mowing + Vegetation height + Vegetation height^2^ + Management intensity^2^xMonth | 15 | 0.00 | 0.376 |
|  |  | 4096 | Bare ground + Bare ground^2^+ Management intensity + Management intensity^2^+ GDD + Grazing + Irrigation + Month + Mowing + Vegetation height + Vegetation height^2^ + Management intensity^2^xMonth | 16 | 1.73 | 0.158 |
|  |  | 2016 | Bare ground + Bare ground^2^+ Management intensity+ Management intensity^2^ + GDD + Irrigation + Month + Mowing + Vegetation height + Vegetation height^2^ | 14 | 1.77 | 0.155 |
| *Orthopteran species richness* |  | 1024 | Bare ground + Bare ground^2^+ Management intensity + Management intensity^2^+ GDD + Grazing + Month + Mowing + Vegetation height + Vegetation height^2^ | 16 | 0.00 | 0.371 |
|  |  | 1008 | Bare ground + Bare ground^2^+ Management intensity + Management intensity^2^+ Grazing + Month + Mowing + Vegetation height + Vegetation height^2^ | 15 | 0.81 | 0.248 |
|  |  | 2048 | Bare ground + Bare ground^2^+ Management intensity + Management intensity^2^+ GDD + Grazing + Month + Mowing + Vegetation height + Vegetation height^2^ + Management intensity^2^xMonth | 17 | 1.65 | 0.163 |

**Figure A.1**


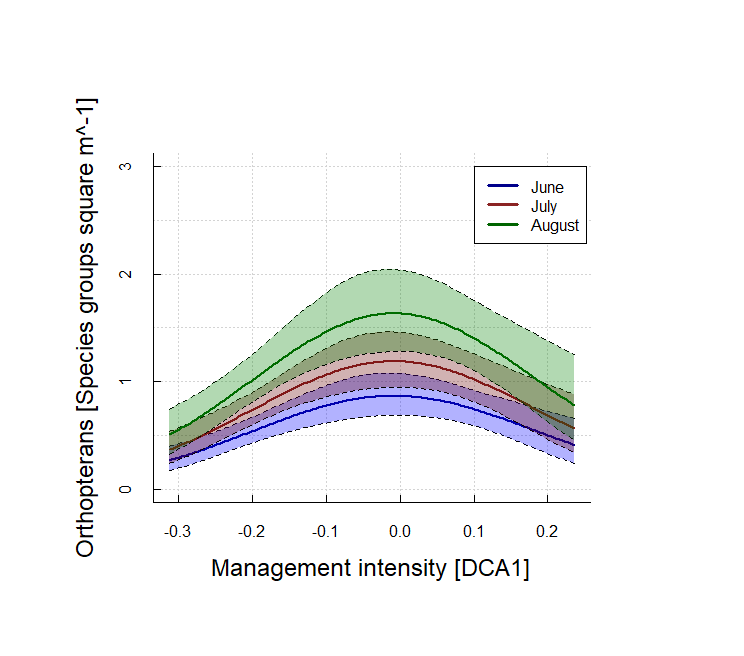
**Figure A.1:** Orthopteran species number in relation to the management intensity index (see Fig.1). The blue curve indicates the response for June, the red for July and the green for August.


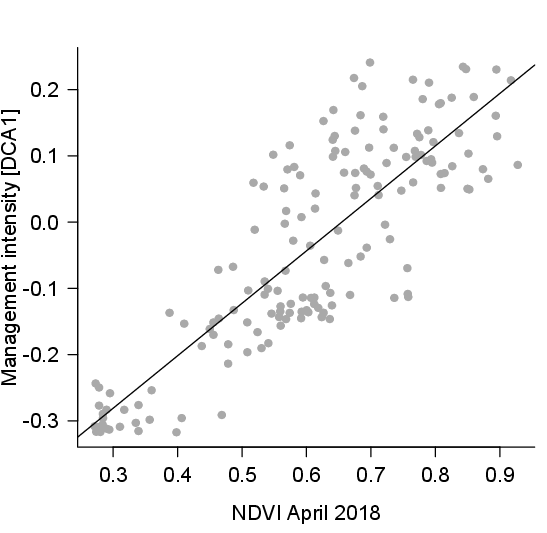
**Figure A.2**

**Figure A.2:** Shows the correlation between the normalized difference vegetation index (NDVI) of April 2018 and the management intensity.

**Figure A.3**


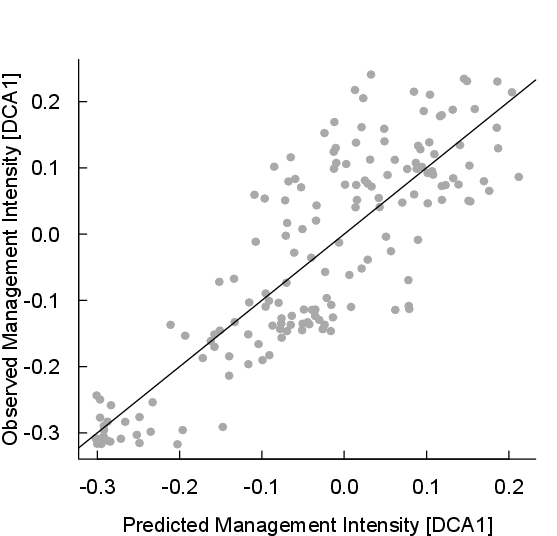

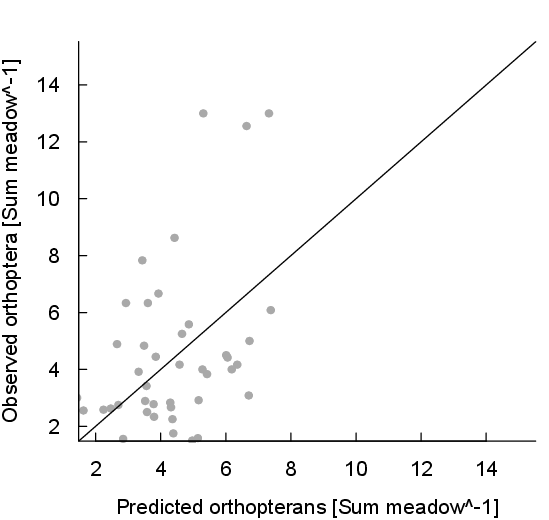
 **a**

**b**

**Figure A.3:** Output of the cross-validation of a) the vegetation proxy projection and b) the projection of orthopteran abundance, showing the predicted values on the x axis against the observed values on the y axis.


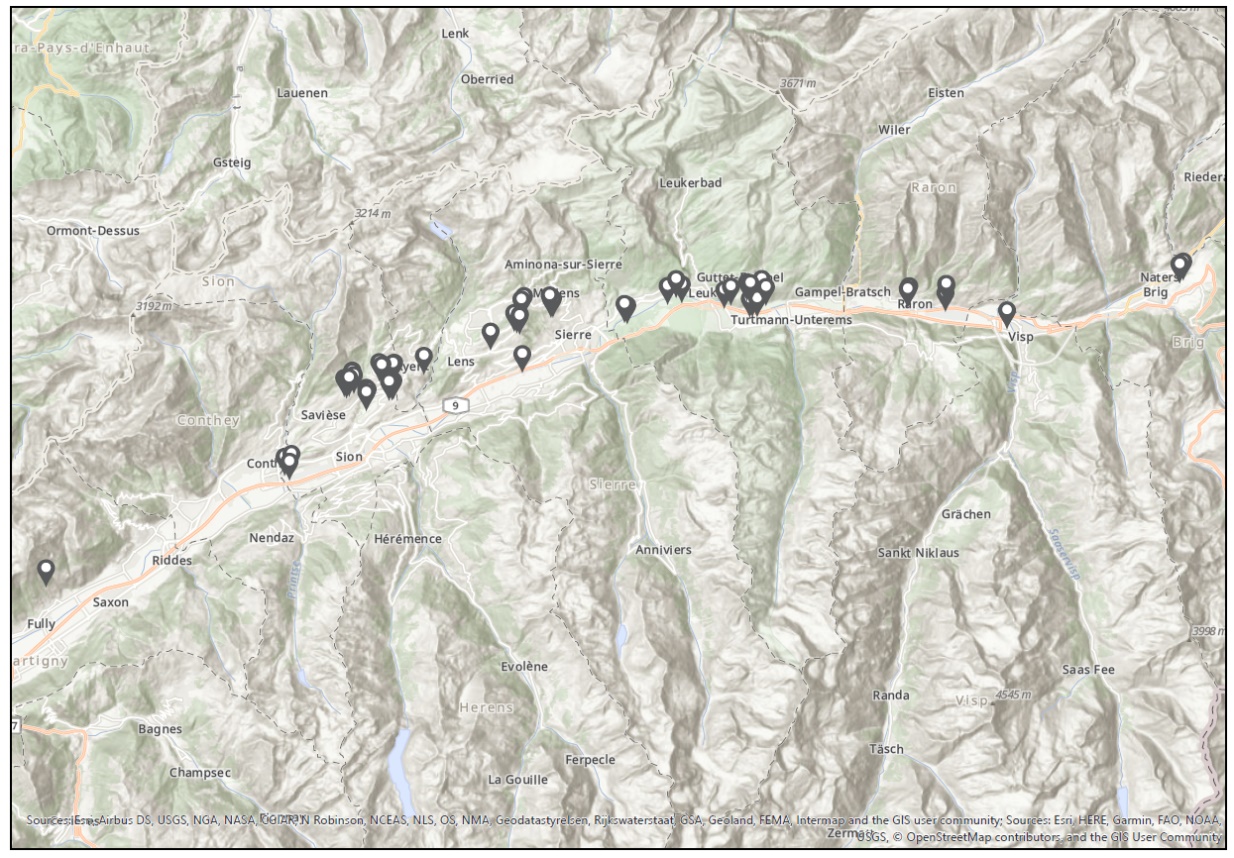
**Figure A.4**

**Figure A.4:** Overview map showing all sampled meadows distributed between Brig and Fully in the Kanton of Valais, Switzerland.

**Figure A.5**


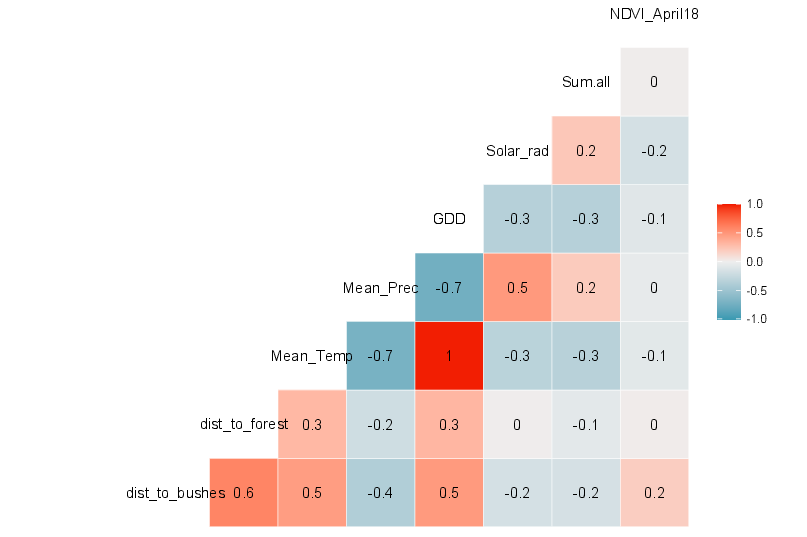
**Figure A.5:** Correlation matrix of non-categorical explanatory variables used in the analyses (see Tab.1 for explanation of the explanatory variables).

**Figure A.6**


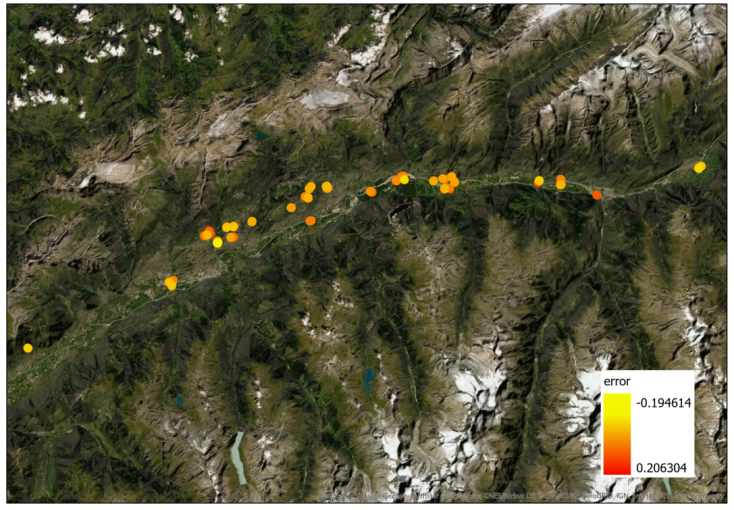

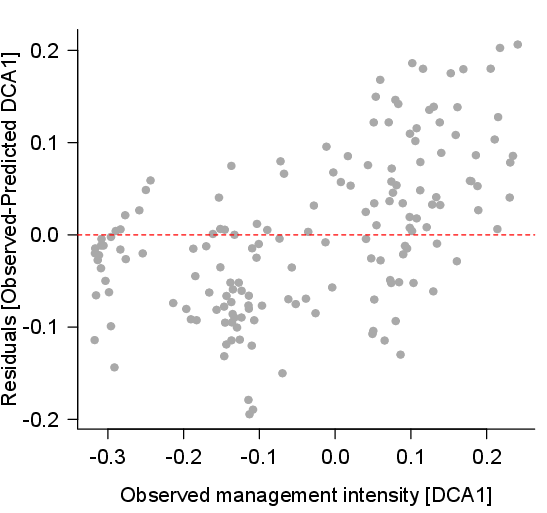
**a**


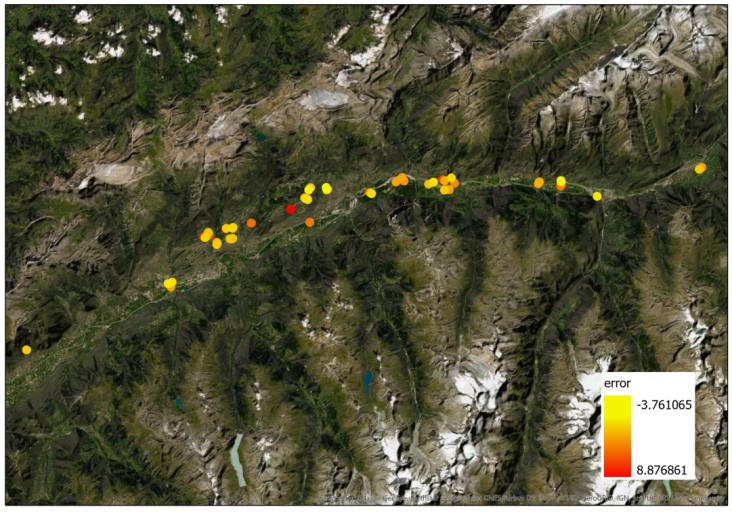

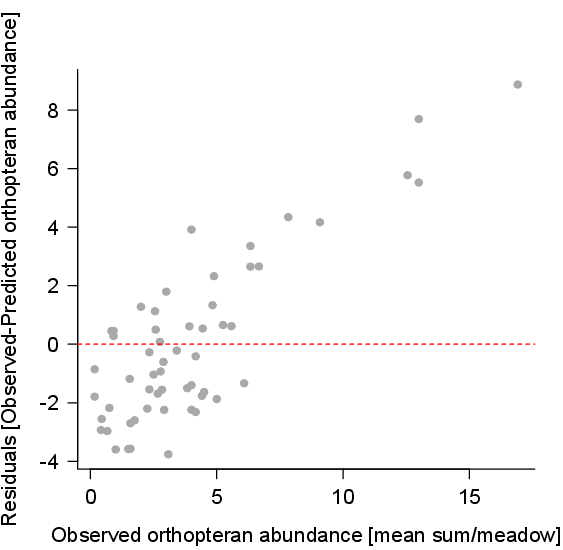
 **b**

**Figure A.6:** Illustration of the error in the projection of a) management intensity and b) orthopteran abundance, showing residuals in relation to the observed values and how they are spatially distributed in the landscape.


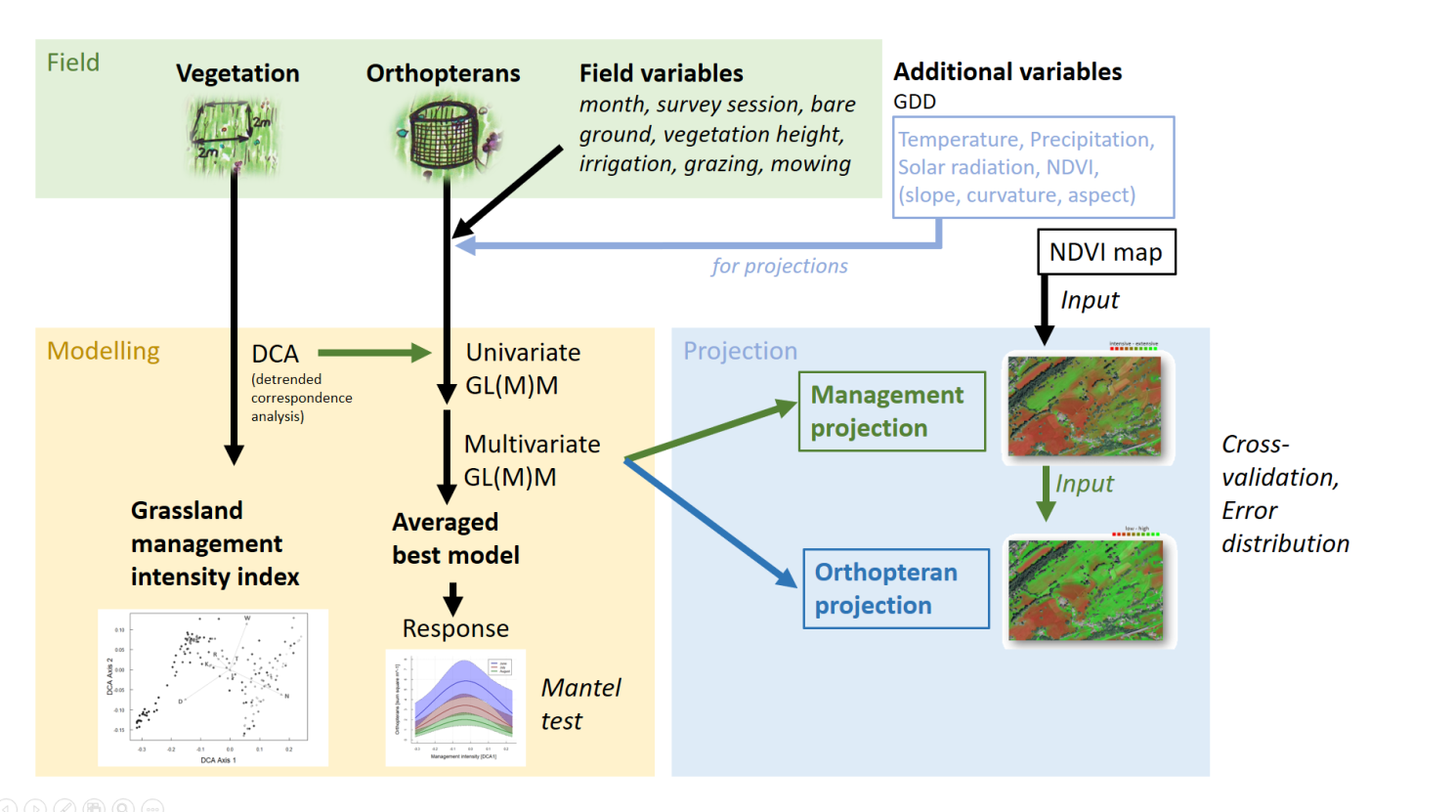
  **Figure A.7**

**Figure A.7:** Diagram showing how the different approaches and models used in the study fit together.
